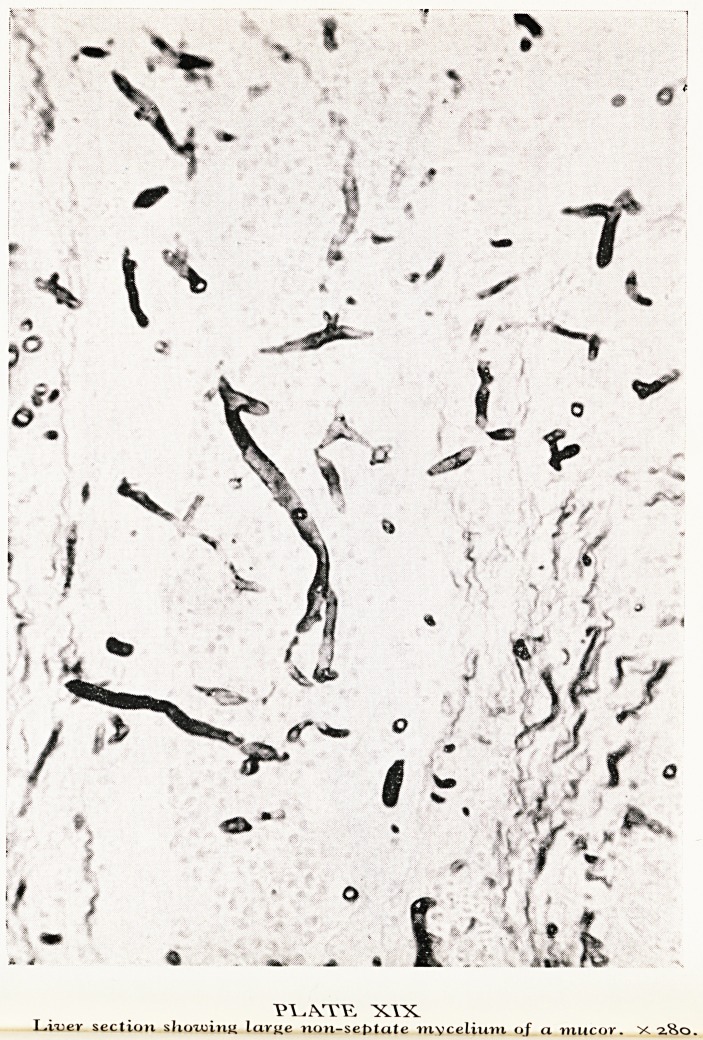# Aspergillosis and Mucormycosis in an Okapi

**Published:** 1964-10

**Authors:** T. F. Hewer


### ASPERGILLOSIS AND MUCORMYCOSIS IN AN OKAPI

*A Clinical-Pathological Conference held in the University of Bristol on* 10th *March* 1964

CHAIRMAN: PROFESSOR T. F. HEWER

*Professor Hewer:* We shall be considering today a recent tragedy in the Bristol Zoo. There is a pair of very beautiful Okapi from Africa which have settled down happily in an enclosure at the Zoo. A male baby was born but fell ill and died. I am going to ask Mr. Packham, a senior Keeper at the Zoo, to tell us what happened.

*Mr. Packham:* The baby okapi was born on 23rd November 1963. It was found at 8.30 in the morning and appeared to be a very strong and healthy baby. During the first day it was seen to feed ten times from the mother, the feeding times varying between 4 and 7 minutes. For the first two days the baby was extremely active in the morning, up to about lunch time; it appeared to rest during the afternoon and spent a fair amount of time sleeping.

From the second day the pattern of behaviour changed somewhat. The baby was active only until about 10.30 in the morning and after this spent the rest of the day resting and sleeping. We took this behaviour to be perfectly normal; it continued from the second day until the day he died, on 12th December. It was found lying on the floor with its head near a drain; it had obviously died in some stress, the straw was considerably disarranged and both the buckets of water we keep in the enclosure had been overturned. The baby was immediately put in the refrigerator and then brought down to the University Department of Pathology.

*Student:* Was the baby suckling and feeding?

*Mr. Packham:* Yes, it was suckling from the mother. The feeds varied each day, they went up to about 7 minutes towards the end of the baby's life.

In point of fact the baby's measurements increased by about 4½ inches round the girth, 3 inches in height and 4 inches in length during the fortnight we had him.

*Professor Hewer:* I did the post-mortem examination. The only external abnormality was a patch on the forehead where some hair had been recently rubbed off: this may well have been due to a convulsion.

When I opened up the body the first finding of importance was in the chest. It had a very striking appearance, the whole surface of all lobes of both lungs was equally involved in hard whitish areas with some hyperaemia around them. I could feel the glands at the hilum of the lung were enlarged. On cutting into the lung I found these pale areas, which I had seen on the surface looking almost like abscesses, had a lobular arrangement, centred round the bronchi and bronchioles like bronchopneumonia. It did not look like tubercle and I was very uncertain what it was. (Plate XVI.) I took material from the lesions and gave it to Dr. Linton who very kindly examined it for us. Would Dr. Linton tell us about his examination of this material?

*Dr. A. H. Linton:* When the lung aspirate was received in the laboratory we expected the cause of the abscess-like areas in the lungs to be pyogenic bacteria. The material, therefore, was cultured on various bacteriological media and also on Sabouraud agar. After 24 hours incubation at 37°C all the inoculated plates revealed heavy growths of *Aspergillus fumigatus* the identity of which was later confirmed by Miss Mary English. Since the lesions in other parts of the body were thought at the time to be caused by the same organism material from these was not submitted for bacteriological examination.

*Professor Hewer:* I took blocks of the lung for histological examination. The white patches proved to be areas of necrosis with exudate within the alveoli. They had the appearance of lesions produced by aspiration into the lung. In these necrotic areas there were many septate, branching mycelia of aspergillus (Plate XVII).

In a section of one of the enlarged tracheal lymph nodes I was surprised to find a rather different organism consisting of larger mycelia with no septa. This organism was not present in the lung tissue but was recognizable in large numbers within the blood vessels at the hilum. I was not, of course, aware of the presence of two kinds of mycelium until some days later, when the sections were ready.

The heart had some patches of inflammatory exudate on the epicardium over some myocardial abscesses that had fresh haemorrhages around them. The photograph (Plate XVIII) shows these haemorrhages and also a dark thrombus attached to the wall of the left atrium and another arising from the wall of the left ventricle at the site of one of the abscesses. There were also some haemorrhages within the substance of the mitral valve.

In the liver and kidneys there were similar abscesses associated with haemorrhage. In the mucosa of the abomasum, the last part of the ruminant stomach, there were some small discoloured ulcers. Sections of all these lesions showed the same picture. They were all characterized by the presence of a non-septate mycelium which was larger than the aspergillus. This organism was present also in the blood vessels of all the tissues examined and the small veins in the submucosa around the ulcers of the abomasum were full of it. The thrombi in the heart were full of it. A photomicrograph of one of the veins in the liver (Plate XIX) shows the mycelia.

It became clear that this was a double infection.

The long lesions were evidently due to inhalation of a great many spores of *Aspergillus fumigatus*, while the portal of entry of the other organism, which was considered by Dr. Linton to be a mucor, was apparently the ulcers in the abomasum. The mucor must have been ingested. The aspergillus was confined to the lungs. I sent some material from these tissues to Mr. J. A. J. Venn, the Veterinary Investigations Officer, at Langford, and he very kindly examined it independently and came to the same conclusion, that is to say, the baby okapi inhaled a great many spores of *Aspergillus fumigatus* and ingested a mucor that gained an entry into the blood stream by invasion of small venules in the submucosa of the abomasum.

The question that now arises is the source of this heavy infection. The most likely answer is that it came from the hay that was fed to the parent okapis or from the bedding. As soon as we discovered the nature of the infection we told Mr. Greed, the Director of the Zoo, and suggested an examination on these lines.

I will ask Mr. Packham, the keeper in charge of the okapis, to tell us about the feeding arrangements.

*Mr. Packham:* The mother was fed on clover hay entirely throughout the period of the baby's life. The litter was just ordinary wheat straw and this was changed every day. It was taken out of the enclosure, and was burnt, and the whole enclosure was thoroughly scrubbed out, and once a week it was burnt all over with a flame. The straw was put out every night.

*Professor Hewer:* The hay was put in one of the racks they feed from?

*Mr. Packham:* Yes.

*Professor Hewer:* We sent samples of the clover hay and of the bedding straw to Mr. Venn at Langford and I will read you his report. "It was found that the hay carried such a burden of fungi as to qualify for a label 'mouldy' based on Graham Lacy's classification. (*J. Gen. Microbiol.* 1963.) The straw appeared of good quality on

PLATE XVI

*Lung, showing lesions of aspergillosis.*

PLATE XVII

*Lung section showing branching septate mycelium of* Aspergillus fumigatus. ×280.

PLATE XVIII

Heart, showing endocarditis, myocardial abscesses and infected mural thrombi.

PLATE XIX

Liver section showing large non-septate mycelium of a mucor. × 280.

direct examination. It did however yield fairly heavy recoveries of aspergillus and mucors. This suggests that it too is not of the highest quality, although perhaps if it has been stored in the same place as the hay there may have been cross contamination from hay to straw."

Mr. Greed, the Zoo Director, had great difficulty in getting really perfect hay last autumn on account of the wet summer; a great deal of hay was spoiled.

I should like to ask Professor T. K. Ewer, the professor of Animal Husbandry, to comment on this feeding stuff problem.

*Professor T. K. Ewer:* It seems to me that if you are going to avoid contamination with fungi you can go about it in two ways: either you can feed something which is very dry, and of course the fungi won't grow in it, or you can feed something which has the sort of pH which would prevent it growing. I am assuming that you must provide plenty of roughage, since okapis are ungulates and require it. You might use silage, which is an acidic material and very frequently fed to ruminants, but I can see a number of objections to doing so in zoos. First of all, it is difficult to store; it smells strongly and I suspect would be found to be unpalatable to some animals. This is one way in which you can certainly avoid trouble with fungi. However, I think a more practical way would probably be to be sure that the roughage you feed is really dry, and today, with forced draught drying of hay becoming commercially applicable, I think that you have a means of avoiding a recurrence of this tragedy. Barn-dried hay, as it is called, is brought down quickly to sufficiently low moisture content to prevent organisms growing. I cannot help feeling that good barn dried hay bought from a careful operator may be a practical answer.

*Professor Hewer:* I don't know whether Mr. Greed, who is here with us tonight, would like to make a comment on this?

*Mr. Greed:* In the first place I think silage would be decidedly off; there is the question of storage and the rather unpleasant smell. As regards the dried grass, I doubt whether we could feed this in sufficient quantity. These animals are feeding practically all day long, and take a large amount of clover hay. We do alternate between clover hay and lucerne; in fact we are using lucerne at the moment; it is better quality than the clover available at the time of the baby's birth; also there is much difficulty in getting dried hay. I understand that it is ground down and used by feeding stuff manufacturers.

*Professor T. K. Ewer:* I think you have to be clear that I am speaking of barn-dried hay bought in the bale, and this is not the same as buying dried grass, which, as you say, is largely used by the feedstuff manufacturers. I have seen several farms that have just started a drying process in this part of the country, so I believe it will be possible to get very good quality, nutritious clover and grass barn-dried hay now, at not very much more than you have to pay for first quality field-dried hay and you can be sure of it being of extremely low moisture content.

*Question:* Does barn dried hay, without clover, have too low a protein content? And in that case can you not give feed concentrates as an addition?

*Mr. Greed:* Yes, we do give feed concentrates. Do not forget too that they take a tremendous amount of evergreen oak leaves at this time of the year and also during the summer months.

*Dr. Arthur:* Do I understand the baby was already taking solids at the age of 3½ weeks?

*Professor Hewer:* No. The mother and father were.

*Question:* I thought the idea was that the baby ate the mucoraceous fungi?

*Professor Hewer:* I think it was the dusty air. If you inhale anything it goes through the mouth, and animals lick their coats.

*Dr. Hunt:* Does the temperature and degree of ventilation of the house make any difference? It is kept at a higher temperature for these animals?

*Mr. Packham:* It is kept at about 60°; it has been since the baby was born. It was well ventilated.

*Question:* Surely this animal would be taking some roughage from about 12 hours old? Calves do.

*Mr. Packham:* The baby was observed completely during the whole of one day, from first thing in the morning until last thing at night, and there was no suggestion that it was picking up anything at all from the ground. The only possibility that he could have taken clover was when the mother fed from the hay rack and it is possible that some of it fell on the floor but I personally did not see the baby pick up any of this. It may of course have done after the zoo was closed.

*Professor T. K. Ewer:* It was 20 days old, wasn't it, when it died? I would have guessed that before this it was trying to get hold of bits of leaf and so on. This would be quite natural.

*Professor Hewer:* It sounds as though it started being ill after about 4 days, so infection must have occurred very early.

*Mr. Packham:* The pattern of behaviour certainly changed.

*Professor T. K. Ewer:* I am sure the aspergillus was inhaled.

*Question:* May I ask what the rumen content showed? This would surely give a guide as to what it had been eating?

*Professor Hewer:* It was empty except for a trace of milk.

*Dr. McConnell:* May I ask whether any other cases of infection with either aspergillus or mucor have occurred in the zoo? And if not how one could account for it?

*Professor Hewer:* Aspergillus spores are everywhere. Given the right conditions an animal is very likely to get the disease.

*Professor T. K. Ewer:* I think it is a measure of their susceptibility. These young animals are extremely susceptible.

*Dr. McConnell:* This was the only death you have had?

*Professor Hewer:* We have aspergillosis in many birds, the penguins particularly. Of course the spore count in the hay was, I understand, extremely high and this is a point where I think we might get advice from Professor Ewer. The hay has to be put in a rack and the animal pulls it down. In so doing it must spread a tremendous amount of dust over the room. Would it be better to have it on the ground?

*Professor Ewer:* No, I think it is better not to have it contaminated by anything on the ground.

*Professor Hewer:* But the contamination is in it already!

*Professor Ewer:* You would not want it mixed up with the straw. I think the rack is right.

*Dr. Linton:* Do you not think it would be possible for the baby to suckle the mother and the udder, teats and so on be contaminated with mucoraceous fungi? It is quite conceivable that the mucor got in with the milk without any thought of whether it was eating solids at all.

*Mr. Greed:* I was coming to the point you have just raised. I would like to point out that these animals spend much time licking themselves; in fact after about 3 or 4 days we observed the baby licking its mother's coat all over.

*Question:* If you diagnosed this condition how would you treat it?

*Professor Hewer:* There is no treatment.

*Question:* Could you not damp the hay so as to reduce dust?

*Professor Ewer:* I should think this would be very difficult. I think it would interfere to some extent with the animal's pattern of feeding.

*Dr. Marrian:* Presumably adult animals are exposed to this all the time. What is is it that determines their susceptibility? Have they developed antibodies or is there a higher level of acid in their stomach which kills off the ingested organism? What makes the difference between this baby and the parents?

*Dr. Lloyd:* I wanted to ask that question as well. Are not either of the parents suffering from either of these diseases, and if not, why not?

*Professor Hewer:* We do know that in the mother's faeces aspergillus spores have been found, but happily she is well and the presence of the spores probably means nothing.

*Dr. Marrian:* She may have been protected from infection by antibodies. Are these known to exist and can they be detected and levels measured in animals?

*Professor Hewer:* Can Miss English answer that? Could they be detected?

*Miss English:* Yes, there is some evidence of detectable antibodies in man, especially in known cases of aspergillosis, but they appear to have little effect on the course of the disease. I know of no similar observations on animals.

*Professor Hewer:* As the organism is ubiquitous I suppose animals all develop antibodies to it eventually, but much must depend on the dose.

*Dr. Partington:* It is well recognized that in the human these fungus conditions, aspergillosis and mucormycosis, occur in people who are debilitated, often with leukaemia or reticulosis, or else in people who are on cortisone or any antibiotic. I was going to suggest that hypogammaglobulinaemia might have occurred in the baby okapi. Might it not be a question of low gamma globulin?

*Dr. Hunt:* Is the transmission of antibodies different in zoo animals? Does this mean that the mother did not have any antibodies?

*Professor T. K. Ewer:* I do not know. I doubt whether we have sufficient knowledge about the antibody titre against fungi.

*Professor Hewer:* I should have thought it would take an awful lot of antibody to deal with all these spores. The dosage must have been colossal and I dare say that the baby, being much lower down on the ground got much more dust in its lungs than the mother or father.

*Question:* Would it be possible to make a routine examination for spores in the fodder?

*Professor Hewer:* Oh, yes. I am sure that next time Mr. Greed will see to it that spores counts are made as far as possible, or at any rate that the hay and straw are very carefully examined. During the wet weather last summer hay, I understand, was extremely difficult to cure.

*Mr. Packham:* If we assume that the adult okapi are now well and that another baby will be born, would it be wise for the first few weeks for the mother to be fed on nothing but really good evergreen and the normal balanced ration, and cut out hay for that period?

*Professor T. K. Ewer:* It is a little difficult of course to include much fresh grass in a ration during November and December.

*Professor Hewer:* It is really a question then of family planning!

*Question:* Is it possible to use sawdust or chippings instead of straw?

*Professor Hewer:* The hay is the source of infection, not the straw.

*Mr. Packham:* The difficulty there would be that when the animals are feeding they very often drop food on the floor and occasionally pick it up and so would get mouthfuls of chippings.

*Dr. R. P. Warin:* A lot of the hay gets mixed up with the straw. We were shown a photograph of the okapi with her baby. In it there is a mixture of hay and straw on the ground. I would have thought something like peat or chippings would be a very good idea.

*Professor Hewer:* All the same, the hay is up in the rack and they are pulling at it while they feed. The dust must have been there all the while.

*Dr. Warin:* It is a question of total dosage, I think. If it can be reduced by avoiding mouldy hay or by reducing the dust it might be successful.

*Question:* We are told that this hay was mouldy. Hundreds of young animals are reared on hay every year, it seems to me that it should be possible to get hold of hay that is not visibly mouldy.

*Mr. Packham:* This was not visibly mouldy.

*Mr. Greed:* This was reasonably good quality hay; I think the situation was aggravated very much by removing the straw bedding frequently. Twice every day this straw was picked up and bagged in the house and removed for burning. A big problem with these animals is reinfestation with internal parasites, and we have to be very careful to try and avoid this. One cannot leave soiled bedding on the ground.

Since the death of this baby I have talked to our friends in the Rotterdam Zoo who have bred these animals and they have had no trouble in this direction although they do feed on lucerne and clover hay too. As regards the bedding, they are very much against using peat because of the animals' habit of constantly licking themselves. They fear that a large intake of peat by mouth would be bad for them. I certainly do not think sawdust would be a good thing to use, neither do I like wood shavings. Woodwool is something that might be considered.

*Professor Hewer:* Thank you very much. I doubt whether we can get any further on this distressing subject.

*Question:* What did it die of? Aspergillosis or mucoraceous infection?

*Professor Hewer:* I think it was really the mucoraceous infection that killed this baby, but it would undoubtedly have died eventually of the pulmonary aspergillosis. I believe the sequence of events was as follows. Aspergillus spores were inhaled in colossal numbers. Mucor spores were ingested in large numbers; these may or may not have caused ulcers in the abomasum. I express some doubt on this point because in young animals acute gastric ulcers are common and they could well provide a portal of entry. Once in the floor of the ulcers the mucor invaded venules and got into the blood stream. Systemic abscesses, in the liver and elsewhere, and endocarditis resulted and I believe this was the cause of death.

## Figures and Tables

**PLATE XVI f1:**
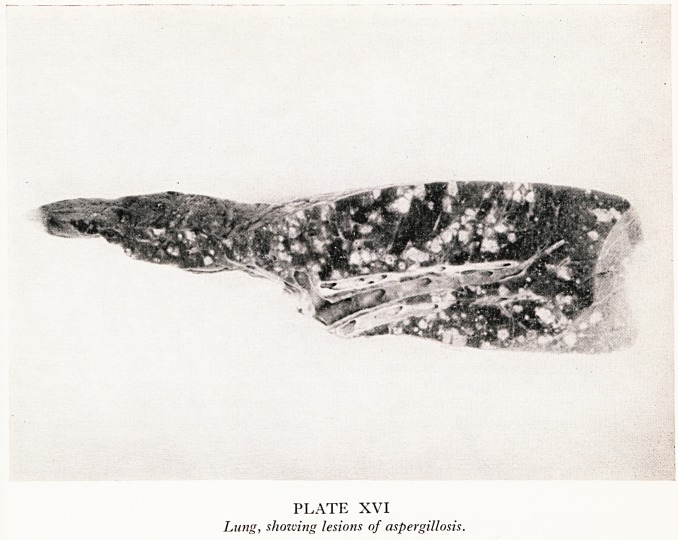


**PLATE XVII f2:**
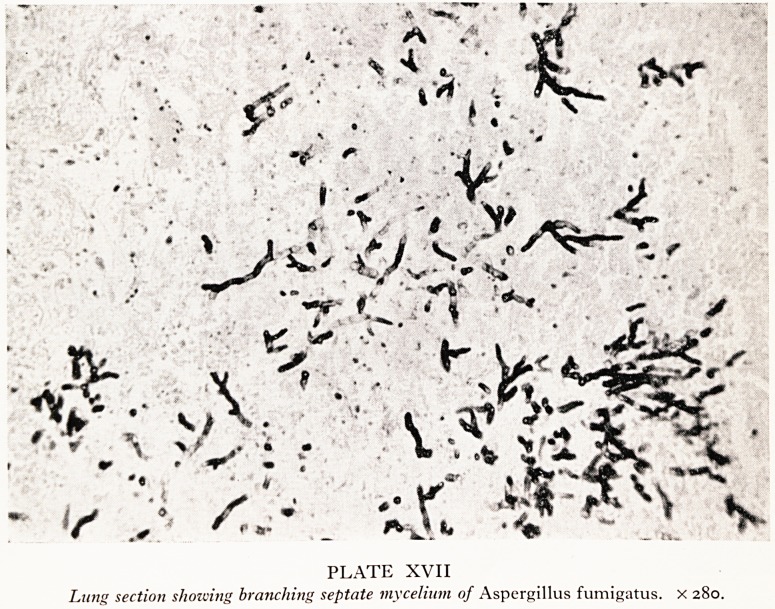


**PLATE XVIII f3:**
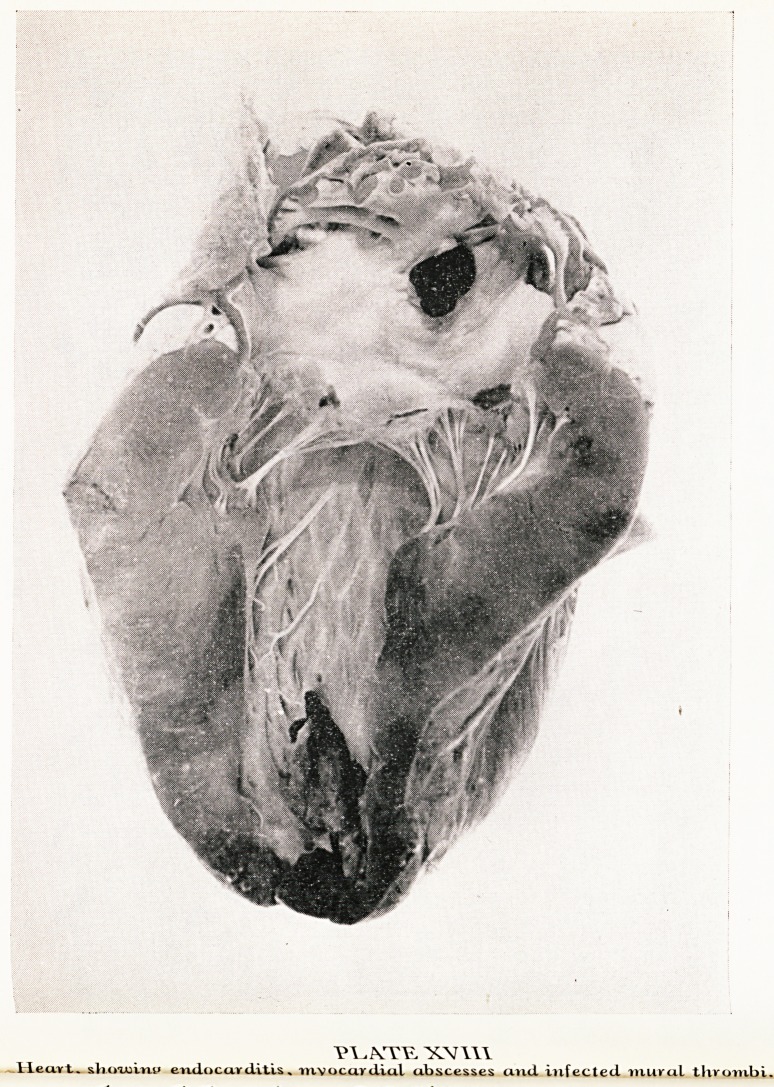


**PLATE XIX f4:**